# Diffusible Compounds Produced by *Hanseniaspora osmophila* and *Gluconobacter cerinus* Help to Control the Causal Agents of Gray Rot and Summer Bunch Rot of Table Grapes

**DOI:** 10.3390/antibiotics10060664

**Published:** 2021-06-02

**Authors:** Matías Olivera, Ninoska Delgado, Fabiola Cádiz, Natalia Riquelme, Iván Montenegro, Michael Seeger, Guillermo Bravo, Wilson Barros-Parada, Romina Pedreschi, Ximena Besoain

**Affiliations:** 1 Escuela de Agronomía, Facultad de Ciencias Agronómicas y de los Alimentos, Pontificia Universidad Católica de Valparaíso, Quillota 2260000, Chile; matias.olivera.a@mail.pucv.cl (M.O.); ninoska.delgado.p@mail.pucv.cl (N.D.); fabiola.cadiz@pucv.cl (F.C.); natalia.riquelme.a@mail.pucv.cl (N.R.); wilson.barros@pucv.cl (W.B.-P.); romina.pedreschi@pucv.cl (R.P.); 2 Escuela de Obstetricia y Puericultura, Facultad de Medicina, Universidad de Valparaíso, Viña del Mar 2520000, Chile; 3 Molecular Microbiology and Environmental Biotechnology Laboratory, Department of Chemistry & Center of Biotechnology Daniel Alkalay Lowitt, Federico Santa María Technical University, Valparaíso 2340000, Chile; michael.seeger@usm.cl (M.S.); bravoc.guillermo@gmail.com (G.B.)

**Keywords:** biocontrol agents, antifungal compounds, fungal growth, in vitro inhibition

## Abstract

Gray and summer bunch rot are important diseases of table grapes due to the high economic and environmental cost of their control with synthetic fungicides. The ability to produce antifungal compounds against the causal agents *Botrytis*, *Aspergillus*, *Penicillium*, and *Rhizopus* of two microorganisms isolated from table grapes and identified as *Hanseniaspora osmophila* and *Gluconobacter cerinus* was evaluated. In dual cultures, both biocontrol agents (together and separately) inhibited in vitro mycelial growth of these pathogens. To identify the compounds responsible for the inhibitory effect, extractions were carried out with organic solvents from biocontrol agents separately. Through dual cultures with pathogens and pure extracts, only the hexane extract from *H. osmophila* showed an inhibitory effect against *Botrytis cinerea*. To further identify these compounds, the direct bioautography technique was used. This technique made it possible to determine the band displaying antifungal activity at Rf = 0.05–0.2. The compounds present in this band were identified by GC-MS and compared to the NIST library. The most abundant compounds, not previously reported, corresponded to alkanes, ketones, alcohols, and terpenoids. *H. osmophila* and *G. cerinus* have the potential to control the causal agents of gray and summer bunch rot of table grapes.

## 1. Introduction

Table grape is the most cultivated fruit species in Chile; its growth area covers 14% of the national fruit industry and the country is positioned as the main exporter worldwide [1]. Two of the most important diseases in table grape production are gray rot (*B. cinerea*) and bunch rot (species of the genus *Aspergillus*, *Botrytis*, *Penicillium* and *Rhizopus*) [2,3]. It is estimated that botryticides represent 10% of the world fungicide market and that the wine and table grape segment represents 50% of the total value of the botryticide market [4]. In Chile, the cost of botryticides amounts to USD 22.4 million year^−1^ [5]. For these reasons, *B. cinerea* is considered the second most important pathogen in the world [6].

It has been of particular scientific interest to investigate methods of controlling table grape bunch rot diseases. Due to the growing public interest in consuming safe food with the least possible environmental impact, restriction of fungicides, maximum residue limits and the frequent development of resistant strains of *B. cinerea* [7,8] to synthetic fungicides, biological products have acquired great importance [9,10,11,12].

Previous works have revealed that micro fissures occur in the cuticle during grape ripening, which is colonized by oxidative or weakly fermentative ascomycetes and basidiomycetes [13]. If the berry has wounds, ascomycetes with high fermentative activity and acetic acid bacteria—transported by drosophilid insects—predominate in the released juice [14,15]. Within the yeasts and bacteria present in this rotting process (called bunch rot, sour rot or ripe rot), the genera *Hanseniaspora* and *Gluconobacter* consistently prevail [13,14,15,16,17]. While the role of the bacteria in berry rot is well known, there are very limited studies that have evaluated their biocontrol activity. Within the framework of development of new “cleaner” technologies, the Phytopathology Laboratory of the Pontificia Universidad Católica de Valparaíso has developed a biological product to control gray and summer bunch rot diseases that affect table grapes. For this, the organisms that intervene in the causative complex of both diseases were isolated, and it was found that two of them show potential to be used as biocontrol agents (BCAs), which correspond to a bacterium, *Gluconobacter cerinus*, and a yeast, *Hanseniaspora osmophila*.

Although there is evidence of the control of fungal diseases in different crops through the use of strains of the genera *Gluconobacter* [18] and *Hanseniaspora* [19,20,21,22,23], their biocontrol mechanisms have been poorly investigated. These mechanisms are the result of a BCA–pathogen–host interaction and classified in production of volatile (VOCs) and diffusible organic compounds, competition for space and nutrients, parasitism, production of lytic enzymes, and resistance induction [24,25]. Unlike VOCs, diffusible compounds are in a liquid or solid state at atmospheric temperature and pressure. Our research group has studied the VOCs produced by *H. osmophila* and *G. cerinus* on the mycelial growth of the causal agents of both diseases [26]. However, the existence of diffusible antifungal compounds is still unknown. Therefore, the following study aims: (i) to evaluate the production and effect of diffusible organic compounds produced by the BCAs on the pathogens causing gray and summer bunch rot of table grape and (ii) to identify the diffusible compounds through GC-MS.

## 2. Results and Discussion

### 2.1. Morphological and Molecular Identification of Pathogens

In this study, the phytopathogenic fungi with which we work were duly identified and sequences were deposited in GenBank as *Botrytis cinerea* (ITS: MT218334 and beta-tubulin: MT228634), *Penicillium expansum* (ITS: MT218335 and beta-tubulin: MT228635), *Aspergillus tubingensis* (ITS: MT218336 and beta-tubulin: MT228636) and *Rhizopus stolonifer* (ITS: MT227125).

### 2.2. In Vitro Effect of Diffusible Compounds Produced by BCAs

**Figure 1:**
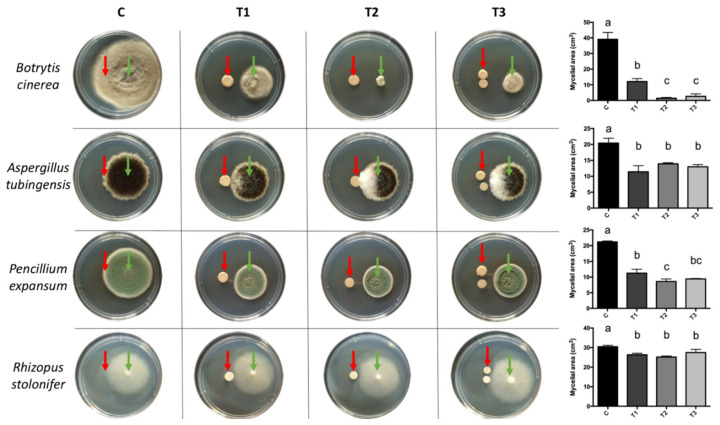
Dual cultures of BCAs (red arrows) with pathogens (green arrows). For all pathogens, an inoculum of 20 µL was used at a concentration of 1 × 10^5^ conidia mL^−1^. The concentration for *G. cerinus* was 1 × 10^6^ CFU mL^−1^ and 1 × 10^4^ cells mL^−1^ for *H. osmophila*. (C) Control: 20 µL of sterile distilled water, (T1) 20 µL of *G. cerinus*, (T2) 20 µL of *H. osmophila*, and (T3) 10 µL of *G. cerinus* and 10 µL of *H. osmophila*. Vertical bars represent standard deviation and different letters indicate significant differences according to Tukey’s test (*p* < 0.05).

The mycelial growth of all pathogens was inhibited by the diffusible compounds produced by the BCAs concerning the control treatment (Figure 1). Mainly, inhibition was more significant against *B. cinerea* and *P. expansum*. The highest percentage of inhibition was presented in the trial with *B. cinerea*, with an average for all treatments of 86.3%. In the case of *P. expansum*, the average inhibition percentage was 53.9%.

Biocontrol activity of *G. cerinus* against *B. cinerea* have been reported [18], but its mode of action was not investigated. Studies that support the biocontrol activity are more numerous in the case of species of the genus *Hanseniaspora* [19,20,21,22,23,27,28,29]. Several authors have evaluated the effect of *H. uvarum* on the gray rot of grapes in vitro and in vivo using the methods of dual culture and co-inoculation in berries. Some of them were able to demonstrate that the yeast was capable of reducing (or inhibiting) spore germination and mycelial growth of the pathogen when applied alone [28] or in combination with adjuvants [20,27,29]. The biocontrol activity of *Hanseniaspora* spp. has been also associated with the induction of resistance in the berry, resulting in a decay reduction, maintenance of fruit quality parameters, and an increase in the synthesis of PR proteins [19,20,29]. Other authors have attributed the effect of biocontrol on possible competition mechanisms [21,22] and production of VOCs [23]. As previously reported, yeasts are promising BCAs as they present several advantages compared to other microorganisms [30,31]. However, a deep understanding of the action mechanism is required to develop appropriate formulation and application methods [24].

### 2.3. Extraction of Diffusible Compounds and Extract Evaluation

In the case of extraction with organic solvents (dichloromethane and hexane) of the supernatant from the BCA cultures, the amount of final solute obtained from the dichloromethane extractions was 28.9 mg (1.45% extraction yield) and 47.4 mg (2.37% extraction yield) for *G. cerinus* and *H. osmophila*, respectively.

**Figure 2:**
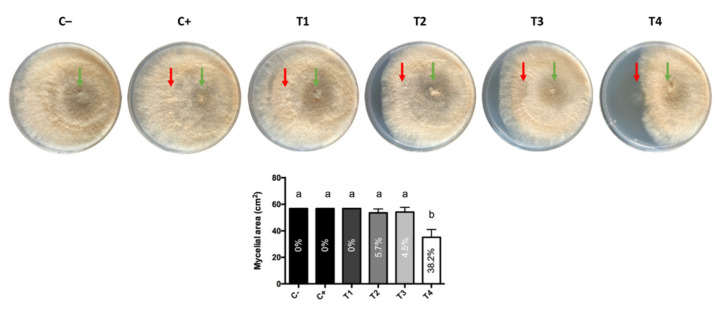
Effect of organic extracts (red arrows) on the mycelial growth of *B. cinerea* (green arrows). For *B. cinerea*, an inoculum of 20 µL was used at a concentration of 1 × 10^5^ conidia mL^−1^. Twenty microliters of each treatment were applied: (C−) negative control (pathogen only), (C+) positive control (20 µL DMSO against the pathogen), (T1) dichloromethane extract of *G. cerinus*, (T2) dichloromethane extract of *H. osmophila*, (T3) hexane extract of *G. cerinus*, and (T4) hexane extract of *H. osmophila*. The percentages inside the bars indicate the percentage of inhibition of *B. cinerea* mycelial growth. Vertical bars represent standard deviation, and different letters indicate significant differences according to Tukey’s test (*p* < 0.05).

Regarding the evaluation of the extracts, treatments based on the dichloromethane extract of *G. cerinus* (T1), the dichloromethane extract of *H. osmophila* (T2) and the hexane extract of *G. cerinus* (T3) inhibited an average of 0, 5.7 and 4.5% mycelial growth of the pathogen, respectively. The only treatment capable of significantly inhibiting the growth of *B. cinerea* was T4 (extract with hexane from the supernatant of *H. osmophila*) with a 38.2% inhibition on average (Figure 2).

### 2.4. Separation and Evaluation of the Extract

**Figure 3:**
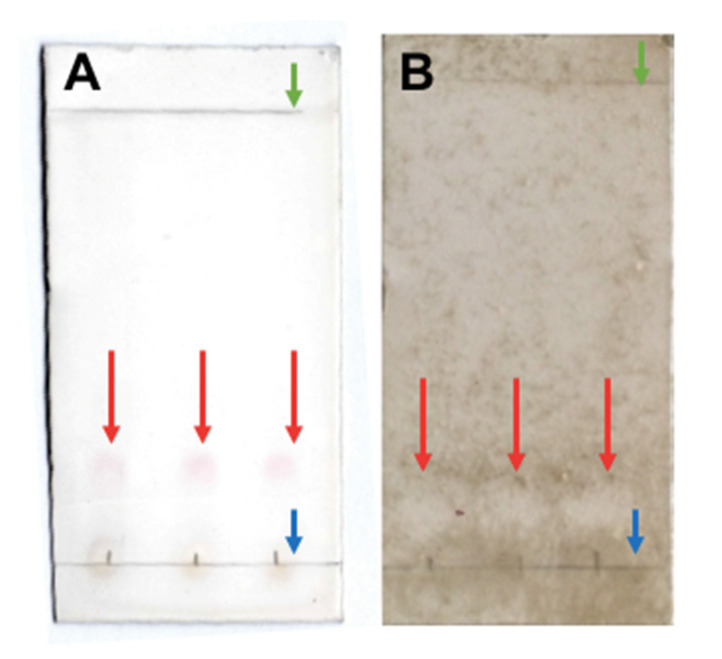
Chromatographic plates of hexane extract from *H. osmophila* culture with mobile phase Hex: EtOAc (6: 1). The chromatographic plates show three lanes as replicates. (**A**) TLC plate developed with 20% H_2_SO_4_ where red arrows indicate revealed compounds; and (**B**) Results for the direct bioautography assay where red arrows indicate growth inhibition of *B. cinerea* at Rf = 0.05 to 0.2. Green arrows indicate the solvent front and blue arrows indicate the starting point.

Development with 20% sulfuric acid (H_2_SO_4_) showed a band near the starting point defined bands (Figure 3A) and bioautography allowed this band, displaying no growth of *B. cinerea* (Figure 3B). Compounds should be mostly non-polar as the retention factor value (Rf) of the band was 0.05 to 0.2. This band was subsequently analyzed by GC-MS to identify the compounds present.

### 2.5. Identification of the Active Compounds of the Extract

GC-MS identification revealed that 29 compounds (66.7% of the total amount present in the sample) were detected in the hexane extract, of which 13 have been reported with antifungal or antibacterial activity in the literature (Table 1). The most abundant group corresponded to alkanes representing 25.5% of the total and correlating with the low migration of compounds in the direct autobiography assay. Previous studies have shown the antifungal activity of tetra- and pentadecane [32] and eicosane and heneicosane [33,34]. Very long-chain alkanes were found to inhibit multiple targets of important pathogenic proteins and enzymes [35]. The second group that appeared in a more significant quantity corresponded to ketones, with 24.1%. 3-methyl-2-butanone [36], 3-pentanone [37], and 4-hydroxy-4-methyl-2-pentanone [38] have also been reported as antifungal compounds, but their mechanism of action remains unknown. Alcohols occupied third place with 15.8% representation. Mannaa and Kim [39] identified 2-isopropyl-5-methylheptanol and 2-butyloctanol in the filtrate of the *Pseudomonas protegens* culture with activity against species of the genera *Aspergillus* and *Penicillium*. Terpenoids were the fourth group, with 15.4% of the total composition. Squalene is the compound that occurred individually in greater abundance in the hexane extract. It is a terpene that contains six isoprene units and is known to be the first specific precursor of ergosterol [40]. Then, the esters followed in fifth place with 6.4%. Huang et al. [41] observed that the propanoic acid ethyl ester or ethyl propionate was produced by *Candida intermedia* and inhibited the mycelial growth of *B. cinerea* by the double-plate method. In sixth place were fatty acids with 4.6%. Zhang et al. [42] identified (Z)-13-docosenamide as one of the four key biocontrol components produced by *Trichoderma longibrachiatum* against *B. cinerea*, *A. niger*, and *R. nigricans*. Likewise, this compound was the most abundant among those produced by a biocontrol strain of *Streptomyces* sp. [43].

On the other hand, Leyva et al. [44] demonstrated the in vitro effectiveness of hexanoic acid in inhibiting the germination of conidia and the mycelial growth of *B. cinerea*, and of reducing the diameter of the lesion caused by the pathogen in tomato plants in a preventive and curative manner. These authors suggested an increase in membrane permeability of the pathogen as an active mechanism. In seventh place with 4.2% was an organophosphorus compound, tris (2-butoxy ethyl) phosphate, which has not been reported in the literature to have antimicrobial or antifungal activity. Finally, benzenes and lactones with 2.2% and 1.9%, respectively. 2,4-di-tert-butyl-phenol has been studied for its antifungal and antioxidant activity [45]. Its modes of action have also been associated with the prevention of spore germination and reduction in mycelial growth [46]. In addition, Raza et al. [47] demonstrated a synergistic effect among the compounds produced by BCAs, thus improving the effect when applying them together.

## 3. Materials and Methods

### 3.1. Morphological and Molecular Identification of Pathogens

The BCAs were obtained from the Phytopathology Laboratory of the Pontificia Universidad Católica de Valparaíso (deposited in the Chilean Collection of Microbial Genetic Resources as *G. cerinus* strain 515, access code RGM2215 and *H. osmophila* strain 337, access code RGM2214). Pathogens were isolated from table grape cv. Red Globe and identified by observing the morphology of colonies (color and texture) and microscopic reproductive structures. For the yeast *H. osmophila*, a HPA medium (80 g L^−1^ honey, 20 g L^−1^ peptone and 20 g L^−1^ agar) was used [48]. *G. cerinus* was cultured and maintained on MYP medium (25 g L^−1^ mannitol, 5 g L^−1^ yeast extract, 3 g L^−1^ peptone and 12 g L^−1^ agar) [49] and PDA (Difco™) was used for all fungal pathogens.

Pathogens were further identified by molecular methods. Sequences were deposited in GenBank as *Botrytis cinerea* (ITS: MT218334 and beta-tubulin: MT228634), *Penicillium expansum* (ITS: MT218335 and beta-tubulin: MT228635), *Aspergillus tubingensis* (ITS: MT218336 and beta-tubulin: MT228636) and *Rhizopus stolonifer* (ITS: MT227125).

### 3.2. In Vitro Effect of Diffusible Organic Compounds Produced by the BCAs on the Pathogenic Fungi

The pathogenic fungi were grown in Petri dishes with PDA medium by seeding a plug of agar with mycelium in active growth. *Rhizopus stolonifer* was incubated at 25 °C for three days; *B. cinerea* at 25 °C for seven days under UV-A light (λ = 350 nm); *Penicillium expansum* and *Aspergillus tubingensis* for seven days at 25 °C. From these plates, the conidia were removed, and a suspension was prepared with sterile distilled water (SDW) at a concentration of 1 × 10^5^ conidia mL^−1^.

Biocontrol agents were grown in Petri dishes in their respective media and incubated at 25 °C for five days. Spores were removed, and a suspension at a concentration of 1 × 10^4^ cells mL^−1^ for the yeast was prepared using a hematocytometer (Neubauer-Impr. Counting chamber, Hirschmann, Germany). Similarly, concentration of the bacterial suspension was adjusted to 1 × 10^6^ CFU mL^−1^ using a spectrophotometer at an OD_580 nm_ (BOECO S-300, Hamburg, Germany).

One centimeter from the center of a PDA plate, 20 µL of the biocontrollers and 20 µL of the pathogens were inoculated in the opposite direction. The treatments consisted of facing each of the BCAs (separately and together) against each pathogenic fungus. As a control treatment, SDW was used against each of the pathogenic fungi. The plates were incubated at 25 °C for three days for *R. stolonifer* and at 25 °C for seven days for the rest of the pathogens. In order to evaluate the experiment, photographs of the plates were taken and the area of the mycelium was measured using the software ImageJ^®^ (NIH, Bethesda, MD, USA). The percentage of inhibition of each treatment was calculated by the following equation: PI = ((CA − TA)/CA) × 100, where PI: percent inhibition; CA: average control treatment; TA: treatment average. The experimental unit was each Petri dish, and the experimental design was completely randomized with three replicates. The experiment was repeated three times.

### 3.3. Extraction of Diffusible Compounds and Evaluation of Extracts

The BCAs were cultured separately using liquid medium sucrose yeast nitrogen (SYN) (20 g L^−1^ sucrose, 10 g L^−1^ yeast extract, and 1 g L^−1^ ammonium chloride, adjusted to pH 5 with HCl and 10 M NaOH) in a 3 L bioreactor (Applikon^®^ Biotechnology, Schiedam, The Netherlands) equipped with a mass flow controller for air, pH and temperature. The bioreactor conditions for the growth of the BCAs were the following: 400 rpm agitation, 1 vvm aeration, pH 5 and 25 °C. The BCAs were previously cultured in Luria–Bertani medium (10 g L^−1^ peptone, 5 g L^−1^ yeast extract and 5 g L^−1^ sodium chloride) and then inoculated at 10% v v^−1^ in 2 L of SYN medium. The culture was harvested in the late exponential phase and centrifuged at 2370 g for 10 min using a centrifuge (Hettich Universal 320, Tuttlingen, Germany) to separate the precipitate from the supernatant. In a separatory funnel, the supernatant was mixed with a stirrer and an organic solvent of low polarity (hexane, Merck LiChrosolv, Darmstadt, Germany) and one of medium polarity (dichloromethane, PanReac Applichem ITW Reagents, Barcelona, Spain) were introduced. Subsequently, the organic solvent was transferred to an evaporation flask and evaporated using a rotary evaporator (Buchi R-300, Zurich, Switzerland) at 30 °C and 200 rpm. To extract the solid fraction from the flask, three milliliters of dichloromethane were added, then the solution was removed with a micropipette and placed in a 50 mL glass jar and left under an extraction hood until the solvent was completely evaporated. Finally, 1 mL of dimethylsulfoxide (DMSO, Merck, Darmstadt, Germany) was added, and the resulting solutions were kept at 4 °C until the new test was started.

To evaluate the effect of the extracts, the dual culture method described above was used, but replacing the BCAs inoculum with its corresponding extract. Additionally, the extracts were evaluated only against *B. cinerea*. The treatments corresponded to 20 µL of each extract: T1) dichloromethane extract of *G. cerinus*, T2) dichloromethane extract of *H. osmophila*, T3) hexane extract of *G. cerinus* and T4) hexane extract of *H. osmophila*. The same volume of DMSO was used as control. Five replicates were used per treatment, and the test was repeated three times.

### 3.4. Separation and Evaluation of the Extract

After the evaluation of extracts, the one with the highest activity (hexane extract from the culture supernatant of *H. osmophila*) was separated by thin-layer chromatography. A silica gel plate (2.5 cm wide and 5 cm long) was used as the stationary phase and a mixture of Hex: EtOAc (6:1) as the mobile phase. As revealers, UV light with a wavelength of 312 nm (Vilber Lourmat ETX-20.M, Germany) and submersion in a solution of 20% sulfuric acid (H_2_SO_4_) followed by drying on a heating plate at 100 °C were used. After separation, the effect of the separated compounds was evaluated by direct bioautography [50]. Briefly, the conidia were extracted from a 7-day PDA plate using a loop previously submerged in Tween 20 and suspended in SDW until reaching a concentration of 1 × 10^7^ conidia mL^−1^. This conidia suspension was mixed 1:100 with PDB to obtain a final concentration of 1 × 10^5^ conidia mL^−1^. Chromatographic plates not revealed with sulfuric acid were immersed in this nutrient suspension and incubated at 25 °C for seven days in a humid chamber. Subsequently, photographs were taken, and the Rf was calculated using ImageJ^®^ software.

### 3.5. Identification of Extract Active Compounds

The band that showed the highest inhibitory effect was scraped from the chromatographic plate and mixed with 1 mL of acetonitrile (LiChrosolv, Merck, Darmstadt, Germany). This solution was separated and its components were identified by a gas chromatograph/mass spectrometry (GCMS-QP2010 Ultra, Shimadzu Corp., Kyoto, Japan). The conditions of the technique were: SLB-5ms capillary column 30 m × 0.25 mm × 0.25 µm (Supelco, Milan, Italy); helium carrier gas flow at 1 mL min^−1^; injection temperature at 250 °C; oven temperature program: 40 °C for 4 min, 10 °C min^−1^ to 270 °C and hold for 10 min and, finally, 10 °C min^−1^ to 290 °C and hold for 10 min; splitless injection at a volume of 1 µL min^−1^ using a Shimadzu AOC-20i auto injector. The compounds of the extract were identified by comparison with the NIST 2014 database (applying > 80% match as acceptance requirement).

### 3.6. Statistical Analysis

Data obtained from the tests were subjected to an analysis of variance (*p* < 0.05) and a Tukey test (*p* < 0.05) was performed in case of significant differences using GraphPad Prism^®^ software (GraphPad Software Inc. v. 6, San Diego, CA, USA).

## 4. Conclusions

The BCAs *Hanseniaspora osmophila* and *Gluconobacter cerinus* were able to inhibit the mycelial growth of all the pathogens used by the production of diffusible organic compounds. The highest percentage of inhibition was observed against *B. cinerea*. Of the extracts used, only hexane extraction from the liquid culture with *H. osmophila* significantly inhibited the growth of *B. cinerea*. By thin-layer chromatography, the components of the extract were separated, and the compounds present in a band with antifungal effects were identified using the bioautography technique. Fifty-five percent of the detected compounds have not been reported as antifungal or antibacterial in the literature. Future work can be conducted to elucidate the role of these molecules in the mycelial growth or spore germination of this pathogen.

## 5. Patents

Patent N° 61580, 7 January 2021, WO2017088081A1.

## Figures and Tables

**Table 1:** Compounds identified by GC-MS from the hexane extract of *H. osmophila*.

N° Peak	RT (Min)	Main Components	RI †	RIref ‡	%Area	Match	Activity §	Reference
1	4.03	2-Butanone, 3-methyl-	590	650	5.11	90	anf	[36]
2	4.13	3-Pentanone	654	672	4.01	91	anf	[37]
3	4.30	Propanoic acid, ethyl ester	686	693	2.08	81	anf	[41]
4	6.60	2-Pentanone, 4-hydroxy-4-methyl-	845	811	6.91	89	anb/anf	[38]
5	10.37	Hexanoic acid	974	950	0.69	92	anf	[44]
6	12.66	Undecane	1115	1100	2.04	93	NR	
7	14.89	Dodecane, 4,6-dimethyl-	1285	1325	0.87	87	NR	
8	15.78	1-Octanol, 2-butyl-	1393	1277	1.88	90	anf	[39]
9	15.91	11-Methyldodecanol	1492	1435	2.76	89	NR	
10	16.03	2-Isopropyl-5-methyl-1-heptanol	1165	1165	2.50	88	anf	[39]
11	17.15	Tetradecane	1413	1400	2.57	98	anb/anf	[32,33]
12	17.90	Hexadecane, 2,6,11,15-tetramethyl-	1753	1792	4.24	84	NR	
13	18.25	Tetradecane, 4-methyl-	1448	1454	0.56	84	NR	
14	18.45	Pentadecane	1512	1500	1.59	97	anb/anf	[32]
15	18.60	2,4-Di-tert-butylphenol	1555	1519	0.95	92	anf	[45]
16	19.02	1-Dodecanol, 2-hexyl-	1989	1504	1.04	81	NR	
17	19.68	Hexadecane	1612	1600	1.67	97	NR	
18	20.84	Octadecane	1810	1800	1.39	94	NR	
19	21.49	1-Heptanol, 2,4-diethyl-	1229	1229	2.33	87	NR	
20	22.93	Eicosane	2009	2000	0.59	82	anb/anf	[34]
21	23.01	2-Methylhexacosane	2656	2656	0.95	83	NR	
22	23.13	1H-Indole-3-ethanol, acetate (ester)	1729	1926	1.70	88	NR	
23	23.18	7,9-Di-tert-butyl-1-oxaspiro(4,5)deca-6,9-diene-2,8-dione	2081	1929	1.23	85	NR	
24	23.99	Heneicosane	2109	2100	0.51	89	anb/anf	[33,34]
25	26.72	Carbonic acid, octadecyl prop-1-en-2-yl ester	2189	2189	0.50	85	NR	
26	27.57	Ethanol, 2-butoxy-, phosphate (3:1)	2363	2363	2.80	94	NR	
27	28.85	Phenol, 2,4-bis(1-methyl-1-phenylethyl)-	2702	2527	0.50	86	NR	
28	32.35	13-Docosenamide, (Z)-	2625	2625	2.38	93	anf	[42,43]
29	32.89	Squalene	2914	2847	10.28	97	NR	

† RI Retention index relative to C8-C36 n-alkanes in a SLB-5ms capillary column; ‡ RIref: Retention index reported in the literature; § Activity reported in the literature: NR (no reference), anf (antifungal) and/or anb (antibacterial).

## References

[1] Larrañaga P., Osores M.A., CIREN-ODEPA (2019). Catastro Frutícola Región del Maule.

[2] Duncan R.A., Stapleton J.J., Leavitt G.M. (1995). Population dynamics of epiphytic mycoflora and occurrence of bunch rots of wine grapes as influenced by leaf removal. Plant Pathol..

[3] Williamson B., Tudzynski B., Tudzynski P., Van Kan J.A.L. (2007). *Botrytis cinerea*: The cause of grey mould disease. Mol. Plant Pathol..

[4] Fillinger S., Walker A.S., Fillinger S., Elad Y. (2015). Chapter 10: Chemical control and resistance management of *Botrytis* diseases. Botrytis—The Fungus, the Pathogen and Its Management in Agricultural Systems.

[5] Esterio M., Auger J., Ramos C., Walker A., Muñoz G., Fillinger S. (2009). *Botrytis* en uva de mesa de exportación: Situación actual de sensibilidad a fungicidas en Chile. Aconex.

[6] Dean R., Van Kan J.A.L., Pretorius Z.A., Hammond-Kosack K.E., Di Pietro A., Spanu P.D., Rudd J.J., Dickman M., Kahmann R., Ellis J. (2012). The Top 10 fungal pathogens in molecular plant pathology. Mol. Plant Pathol..

[7] Latorre B.A., Spadaro I., Rioja M.E. (2002). Occurrence of resistant strains of Botrytis cinerea to anilinopyrimidine fungicides in table grapes in Chile. Crop Prot..

[8] Latorre B.A., Torres R. (2012). Prevalence of isolates of *Botrytis cinerea* resistant to multiple fungicides in Chilean vineyards. Crop Prot..

[9] Aoki T., Aoki Y., Ishiai S., Otoguro M., Suzuki S. (2017). Impact of *Bacillus cereus* NRKT on grape ripe rot disease through resveratrol synthesis in berry skin. Pest Manag. Sci..

[10] Carbó A., Torres R., Usall J., Marín A., Chiralt A., Teixidó N. (2019). Novel film-forming formulations of the biocontrol agent Candida sake CPA-1: Biocontrol efficacy and performance at field conditions in organic wine grapes. Pest Manag. Sci..

[11] Calvo-Garrido C., Viñas I., Elmer P.A., Usall J., Teixidó N. (2014). Suppression of *Botrytis cinerea* on necrotic grapevine tissues by early-season applications of natural products and biological control agents. Pest Manag. Sci..

[12] Rotolo C., De Miccolis Angelini R.M., Dongiovanni C., Pollastro S., Fumarola G., Di Carolo M., Perrelli D., Natale P., Faretra F. (2018). Use of biocontrol agents and botanicals in integrated management of *Botrytis cinerea* in table grape vineyards. Pest Manag. Sci..

[13] Barata A., Malfeito-Ferreira M., Loureiro V. (2012). The microbial ecology of wine grape berries. Int. J. Food Microbiol..

[14] Barata A., Santos S.C., Malfeito-Ferreira M., Loureiro V. (2012). New Insights into the Ecological Interaction Between Grape Berry Microorganisms and *Drosophila* Flies During the Development of Sour Rot. Microb. Ecol..

[15] Barata A., Malfeito-Ferreira M., Loureiro V. (2012). Changes in sour rotten grape berry microbiota during ripening and wine fermentation. Int. J. Food Microbiol..

[16] Mateo E., Torija M.J., Mas A., Bartowsky E.J. (2014). Acetic acid bacteria isolated from grapes of South Australian vineyards. Int. J. Food Microbiol..

[17] Valera M.J., Laich F., González S.S., Torija M.J., Mateo E., Mas A. (2011). Diversity of acetic acid bacteria present in healthy grapes from the Canary Islands. Int. J. Food Microbiol..

[18] Guzzon R., Franciosi E., Larcher R. (2014). A new resource from traditional wines: Characterization of the microbiota of “vino santo” grapes as a biocontrol agent against botrytis cinerea. Eur. Food Res. Technol..

[19] Cai Z., Yang R., Xiao H., Qin X., Si L. (2015). Effect of preharvest application of *Hanseniaspora uvarum* on postharvest diseases in strawberries. Postharvest Biol. Technol..

[20] Qin X., Xiao H., Xue C., Yu Z., Yang R., Cai Z., Si L. (2015). Biocontrol of gray mold in grapes with the yeast *Hanseniaspora uvarum* alone and in combination with salicylic acid or sodium bicarbonate. Postharvest Biol. Technol..

[21] Li W., Zhang H., Li P., Apaliya M.T., Yang Q., Peng Y., Zhang X. (2016). Biocontrol of postharvest green mold of oranges by *Hanseniaspora uvarum* Y3 in combination with phosphatidylcholine. Biol. Control.

[22] Prendes L.P., Merín M.G., Fontana A.R., Bottini R.A., Ramirez M.L., Morata de Ambrosini V.I. (2018). Isolation, identification and selection of antagonistic yeast against *Alternaria alternata* infection and tenuazonic acid production in wine grapes from Argentina. Int. J. Food Microbiol..

[23] Qin X., Xiao H., Cheng X., Zhou H., Si L. (2017). *Hanseniaspora uvarum* prolongs shelf life of strawberry via volatile production. Food Microbiol..

[24] Spadaro D., Droby S. (2016). Development of biocontrol products for postharvest diseases of fruit: The importance of elucidating the mechanisms of action of yeast antagonists. Trends Food Sci. Technol..

[25] Köhl J., Kolnaar R., Ravensberg W.J. (2019). Mode of action of microbial biological control agents against plant diseases: Relevance beyond efficacy. Front. Plant Sci..

[26] Delgado N., Olivera M., Cádiz F., Montenegro I., Madrid A., Bravo G., Fuentealba C., Pedreschi R., Salgado E., Besoain X. Volatile Organic Compounds (VOCs) produced by *Gluconobacter cerinus* and *Hanseniaspora osmophila* displaying control effect against table grape-rot pathogens. Proceedings of the XXVII Congreso SOCHIFIT.

[27] Liu H.M., Guo J.H., Cheng Y.J., Liu P., Long C.A., Deng B.X. (2010). Inhibitory activity of tea polyphenol and *Hanseniaspora uvarum* against *Botrytis cinerea* infections. Lett. Appl. Microbiol..

[28] Liu H.M., Guo J.H., Cheng Y.J., Luo L., Liu P., Wang B.Q., Deng B.X., Long C.A. (2010). Control of gray mold of grape by *Hanseniaspora uvarum* and its effects on postharvest quality parameters. Ann. Microbiol..

[29] Liu H.M., Guo J.H., Luo L., Liu P., Wang B.Q., Cheng Y.J., Deng B.X., Long C.A. (2010). Improvement of *Hanseniaspora uvarum* biocontrol activity against gray mold by the addition of ammonium molybdate and the possible mechanisms involved. Crop Prot..

[30] Nadai C., Fernandes Lemos W.J., Favaron F., Giacomini A., Corich V. (2018). Biocontrol activity of *Starmerella bacillaris* yeast against blue mold disease on apple fruit and its effect on cider fermentation. PLoS ONE.

[31] Parafati L., Vitale A., Restuccia C., Cirvilleri G. (2015). Biocontrol ability and action mechanism of food-isolated yeast strains against *Botrytis cinerea* causing post-harvest bunch rot of table grape. Food Microbiol..

[32] Yuan J., Raza W., Shen Q., Huang Q. (2012). Antifungal activity of *Bacillus amyloliquefaciens* NJN-6 volatile compounds against Fusarium oxysporum f. sp. cubense. Appl. Environ. Microbiol..

[33] Boussaada O., Saidana D., Chriaa J., Chraif I., Mahjoub M.A., Mighri Z., Daami M., Helal A.N. (2008). Chemical composition and antimicrobial activity of volatile components of *Scorzonera undulata*. J. Essent. Oil Res..

[34] Kordali S., Cakir A., Akcin T.A., Mete E., Akcin A., Aydin T., Kilic H. (2009). Antifungal and herbicidal properties of essential oils and n-hexane extracts of *Achillea gypsicola* Hub-Mor. and *Achillea biebersteinii* Afan. (Asteraceae). Ind. Crops Prod..

[35] Bordoloi M., Saikia S., Bordoloi P.K., Kolita B., Dutta P.P., Bhuyan P.D., Dutta S.C., Rao P.G. (2017). Isolation, characterization and antifungal activity of very long chain alkane derivatives from *Cinnamomum obtusifolium*, *Elaeocarpus lanceifolius* and *Baccaurea sapida*. J. Mol. Struct..

[36] Garbeva P., Hordijk C., Gerards S., de Boer W. (2014). Volatiles produced by the mycophagous soil bacterium *Collimonas*. FEMS Microbiol. Ecol..

[37] Qadri M., Deshidi R., Shah B.A., Bindu K., Vishwakarma R.A., Riyaz-Ul-Hassan S. (2015). An endophyte of *Picrorhiza kurroa* Royle ex. Benth, producing menthol, phenylethyl alcohol and 3-hydroxypropionic acid, and other volatile organic compounds. World J. Microbiol. Biotechnol..

[38] Zhang J.W., Li S.K., Wu W.J. (2009). The main chemical composition and in vitro antifungal activity of the essential oils of *Ocimum basilicum* Linn. var. pilosum (Willd.) Benth. Molecules.

[39] Mannaa M., Kim K.D. (2018). Biocontrol activity of volatile-producing *Bacillus megaterium* and *Pseudomonas protegens* against *Aspergillus* and *Penicillium* spp. predominant in stored rice grains: Study II. Mycobiology.

[40] Mantzouridou F., Naziri E., Tsimidou M.Z. (2009). Squalene versus ergosterol formation using *Saccharomyces cerevisiae*: Combined effect of oxygen supply, inoculum size, and fermentation time on yield and selectivity of the bioprocess. J. Agric. Food Chem..

[41] Huang R., Li G.Q., Zhang J., Yang L., Che H.J., Jiang D.H., Huang H.C. (2011). Control of postharvest Botrytis fruit rot of strawberry by volatile organic compounds of *Candida intermedia*. Phytopathology.

[42] Zhang S., Xu B., Zhang J., Gan Y. (2018). Identification of the antifungal activity of *Trichoderma longibrachiatum* T6 and assessment of bioactive substances in controlling phytopathgens. Pestic. Biochem. Physiol..

[43] Qi D., Zou L., Zhou D., Chen Y., Gao Z., Feng R., Zhang M., Li K., Xie J., Wang W. (2019). Taxonomy and broad-spectrum antifungal activity of *Streptomyces* sp. SCA3-4 isolated from rhizosphere soil of *Opuntia stricta*. Front. Microbiol..

[44] Leyva M.O., Vicedo B., Finiti I., Flors V., Del Amo G., Real M.D., García-Agustín P., González-Bosch C. (2008). Preventive and post-infection control of Botrytis cinerea in tomato plants by hexanoic acid. Plant Pathol..

[45] Varsha K.K., Devendra L., Shilpa G., Priya S., Pandey A., Nampoothiri K.M. (2015). 2,4-Di-tert-butyl phenol as the antifungal, antioxidant bioactive purified from a newly isolated *Lactococcus* sp. Int. J. Food Microbiol..

[46] Dharni S., Gupta S., Maurya A., Samad A., Srivastava S.K., Sharma A., Patra D.D. (2014). Purification, Characterization and In vitro Activity of 2,4-di-tert-butylphenol from *Pseudomonas monteilii* PsF84: Conformational and Molecular Docking Studies. J. Agric. Food Chem..

[47] Raza W., Ling N., Yang L., Huang Q., Shen Q. (2016). Response of tomato wilt pathogen *Ralstonia solanacearum* to the volatile organic compounds produced by a biocontrol strain *Bacillus amyloliquefaciens* SQR-9. Sci. Rep..

[48] Dhingra O.D., Sinclair J.B. (1995). Basic Plant Pathology Methods.

[49] Garrity G.M. (2005). Bergey’s Manual of Systematic Bacteriology.

[50] Choma I.M., Grzelak E.M. (2011). Bioautography detection in thin-layer chromatography. J. Chromatogr. A.

